# Neurogenic bladder and neuroendocrine abnormalities in Pol III-related leukodystrophy

**DOI:** 10.1186/s12883-015-0283-7

**Published:** 2015-03-04

**Authors:** Ana Potic, Vera Popovic, Jelena Ostojic, Sandra Pekic, Dusko Kozic, Kether Guerrero, Raphael Schiffmann, Geneviève Bernard

**Affiliations:** Clinic for Child Neurology and Psychiatry, Department of Neurology, Medical Faculty University of Belgrade, 6A Dr. Subotica Street, Belgrade, 11000 Serbia; Institute of Endocrinology, Diabetes and Metabolic Diseases, Department of Neuroendocrinology, Medical Faculty University of Belgrade, 13 Dr. Subotica Street, Belgrade, 11000 Serbia; Clinical Center of Vojvodina, Department of Radiology, Medical Faculty University of Novi Sad, 1-9 Hajduk Veljkova Street, Novi Sad, 21000 Serbia; Oncology Institute of Vojvodina, Department of Radiology, Medical Faculty University of Novi Sad, 4 Put Dr. Goldmana Street, Novi Sad, 21000 Serbia; Departments of Pediatrics, Neurology and Neurosurgery, Montreal Children’s Hospital, McGill University Health Center, 2300 Rue Tupper, Montreal, QC H3H 1P3 Canada; Institute of Metabolic Disease, Baylor Research Institute, 3812 Elm Street, Dallas, 75226 TX USA

**Keywords:** 4H leukodystrophy, *POLR3A*, Neurogenic bladder, Prolactin

## Abstract

**Background:**

Pol III-related leukodystrophies, including 4H leukodystrophy, are recently recognized disorders that comprise hypomyelination and various neurologic and non-neurologic clinical manifestations. We report the unique neurologic presentation of the micturition dysfunction in Pol III-related leukodystrophy and describe the novel endocrine abnormalities in this entity.

**Case presentation:**

A 32-year-old Caucasian female exhibited chronic urinary incontinence that commenced at the age of 7 years and remained the unexplained symptom more than two decades before the onset of progressive neurologic decline. A transient growth failure and absent sexual development with hypoprolactinemia appeared in the meanwhile. Neurologic, endocrine, neuroradiologic, and genetic evaluation performed only in the patient’s thirties, confirmed the diagnosis of 4H leukodystrophy as the only cause of the micturition disturbance.

**Conclusion:**

The report shows for the first time that an unexplained chronic bladder dysfunction should be evaluated also as a possible 4H leukodystrophy, thus alerting to the unexpected neurologic and endocrine features in 4H leukodystrophy.

## Background

4H leukodystrophy is a rare disorder that includes hypomyelination, hypogonadotropic hypogonadism and dental developmental anomalies [1]. It belongs to a spectrum of clinically and radiologically overlapping diseases caused by recessive mutations in either *POLR3A* or *POLR3B*, and collectively designated as “Pol III-related leukodystrophies” [1]. Despite the diversity of neurologic manifestations observed in these entities [1], little is known about sphincter dysfunctions and endocrine features [1]. Impairment of bladder/bowel function may be a part of the clinical picture of leukodystrophies. However, with only few exceptions among adult-onset leukodystrophies, it is neither a prominent nor an early feature of the disease, but rather appears in the advanced stages. According to the current knowledge on Pol III-related leukodystrophies, such evolution of the symptoms seems to be the characteristic of these rare diseases as well. Therefore, the childhood-onset neurogenic bladder persisting for decades as the only neurologic manifestation of Pol III-related leukodystrophy represents a noteworthy, so far unique phenomenon. The data on the accompanying endocrine features are still incomplete in a large number of patients with Pol III-related leukodystrophy due to the deficient endocrine evaluation in this disorder. Apart from the variable observations on the growth hormone deficiency provided in some [2], the main attention has been given to hypogonadotropic hypogonadism in most of these patients. The report broadens the endocrine manifestations in 4H leukodystrophy with regards to the prolactin deficiency and suggests establishing the endocrine protocol for Pol III-related leukodystrophy.

## Case presentation

**Figure 1:**
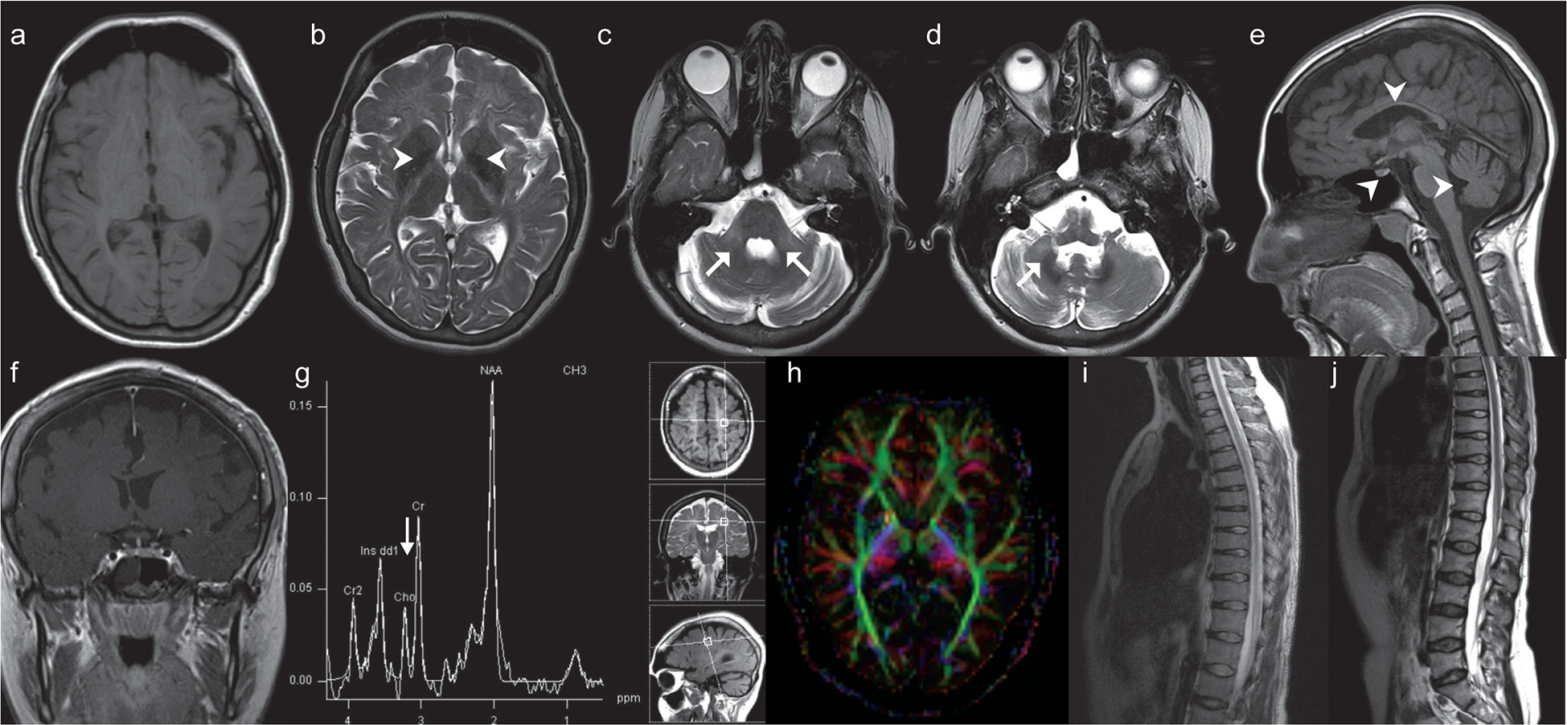
**Magnetic resonance imaging (MRI) study in the patient with 4H leukodystrophy. (a)** T1W (T1-weighted) axial scan of the brain indicating hypomyelination; **(b)** T2W (T2-weighted) scan of the brain and T2W-hypointensity of the globi pallidi; **(c)** affection of the middle cerebellar peduncles:T2W scan; **(d)** T2W-hypointensity of the nucleus dentatus; **(e)** atrophy of the cerebellum, brainstem, corpus callosum and the adenohypophisis: sagittal T1W scan; **(f)** the small adenohypophysis: coronal T1W scan + contrast; **(g)** Magnetic resonance spectroscopy: decreased choline/creatine ratio in the lesions; **(h)** Diffusion tensor imaging of the brain supporting hypomyelination; **(i, j)** the unaffected spinal cord: T2W scans.

The patient was a 32-year-old Caucasian female, the third-born to healthy non-consanguineous parents with negative family history of neurologic disorders. The pregnancy, birth and early psychomotor development were uneventful. At the age of 7 years chronic urinary incontinence commenced and remained the unexplained clinical abnormality for 20 years. Prompt nephrologic, gynecologic, and urologic investigations (also including voiding cystourethrogram, cystoscopy, renal scintigraphy, ultrasonography, biochemical analyses of blood and urine) were normal, and there were no accompanying neurologic signs in the overall clinical status. The persisting urinary disturbance brought the patient to neurologic attention only at 32 years of age when the assessment disclosed a 5-year history of slowly progressive cerebellar ataxia followed by pyramidal signs, and then focal upper extremity dystonia. The neuropsychological evaluation at the age of 32 years documented a mild intellectual disability for the first time (full-scale IQ of 57; Wechsler Adult Intelligence Scale-IV). It was thought to be a decline compared to the patient’s historical level of functioning: the patient had finished the compulsory education (age 7–15 years) with regular psychological assessments, was employed as a factory worker, and was able to live independently. She stopped working at the age of 32 years during the neurologic follow-ups. Magnetic resonance imaging (MRI) of the brain revealed diffuse supratentorial hypomyelination that bilaterally spread along the posterior limb of the internal capsule affecting the pyramidal tracts, the middle and inferior cerebellar peduncles (Figure 1a, b, c). T2-weighted hypointensities were observed in the globi pallidi, the optic radiations and the dentate nuclei (Figure 1b, d). There was a marked atrophy of the cerebellum and corpus callosum, as well as of the pontine and midbrain tegmentum (Figure 1e). The adenohypophysis was small (Figure 1e, f). Multivoxel MR Spectroscopy of the brain detected a markedly decreased choline/creatine ratio in the abnormal white matter (Figure 1g). Diffusion tensor imaging of the brain showed a slightly reduced fractional anisotropy consistent with hypomyelination (Figure 1h). MRI of the brain pointed to Pol III-related leukodystrophy [3]. MRI of the spinal cord was normal (Figure 1i, j). Electromyoneurography was unremarkable. Urodynamic tests (uroflow study, postvoid residual volume, filling-voiding cystometry, abdominal leak-point pressure, external sphincter electromyography-EMG) revealed decreased bladder capacity with preserved sensation, uninhibited detrusor muscle contractions, and detrusor- external sphincter synergy, while sphincter EMG recorded normal amplitudes and duration of muscle potentials. The urodynamic study proved the bladder overactivity due to neurologic, suprapontine lesion [4]. The repeated urologic, nephrologic, and metabolic investigations remained normal. At 26 years of age the patient had no sexual development (Tanner stage 1, primary amenorrhea). Hypogonadotropic hypogonadism, low baseline prolactin level and growth hormone (GH) deficiency were confirmed (Table 1). Her height was below the 3^rd^ percentile and she was normosmic. The karyotype was normal. The patient received sexual hormone replacement for 3 years. At 32 years of age hypogonadotropic hypogonadism was proven by the absence of luteinizing hormone pulsatility (Table 1). Hypoprolactinemia was confirmed by two stimulatory tests (insulin tolerance test-ITT and thyrotropin-releasing hormone-TRH test), while growth hormone level was normal (Table 1). Other hormonal values were normal (Table 1). Her final adult height was 155 cm (10^th^ percentile) and Tanner stage was 4, with amenorrhea. Given the accompanied hypoestrogenemia, gynecologic and urogynecologic examinations excluded atrophic vaginitis, vulvar structural anomalies, and decreased strength of the pelvic floor and bladder muscles, as possible estrogen-related causes of the urinary incontinence [5]. Dental examination confirmed hypodontia with the lack of second and third molars. A genetic evaluation was necessitated: sequencing of the key exons and exon-intron boundaries of *POLR3A* using previously reported methods [6], revealed two already known disease-causing mutations, c.272C > T (p.P91L) in exon 3 and c.3014G > A (p.R1005H) in exon 23 which segregated in the parents and proved the diagnosis of 4H leukodystrophy.

**Table 1:** **Endocrine evaluation in the patient with 4H leukodystrophy**

**Analysis**	**At age of 26 yrs**	**At age of 32 yrs**	**Normal range**
FSH (IU/L)	1.1	1.4	2.5-15
Peak FSH after LHRH test (IU/L)	1.5	1.5	>5
LH (IU/L)^**a**^	<1.0	1.1	4-20
Peak LH after LHRH test (IU/L)	<1.0	1.3	>5
Estradiol (pmol/L)	46.3	13.1	105-217
Prolactin (mean, daily curve, ng/mL)^**b**^	1.5	2.2	4.8-23.4
Peak prolactin after ITT (ng/mL)^**c**^	4.7	4.5	**>> > b**
Peak prolactin after TRH test (ng/mL)^**d**^	N/A	6	**>> > b**
Thyroxine (nmol/L)	134.7	N/A	60-170
Free thyroxine (pmol/L)	N/A	14.1	7-18
Triiodothyronine (nmol/L)	N/A	1.4	0.9-2.4
Free triiodothyronine (pmol/L)	N/A	3.8	2.62-5.7
TSH (mIU/L)	1.0	1.7	0.15-5.0
Cortisol (nmol/L)	332	428	131-642
Peak cortisol after ITT (nmol/L)^**c**^	922	827	>550
IGF-1 (nmol/L)	N/A	26.5	15.1-40.2
Growth hormone (daily curve, μg/L)^**e**^	11.5…0.1…0.1	1.6…0.2…0.4	<0.1
Peak GH after ITT (μg/L)^**c**^	2.4	6.1	>3

*Abbreviations:* *FSH*, follicle-stimulating hormone; *LHRH*, luteinizing hormone-releasing hormone; *LH*, luteinizing hormone; *ITT*, insulin tolerance test; *TRH*, thyrotropin-releasing hormone; *IGF-1*, insulin-like growth factor 1; *GH*, growth hormone.

^**a**^LH measured every 15 minutes during 8 hours; ^**b**^Mean of three diurnal values measured at 08 h-11 h-13 h; ^**c**^After insulin tolerance test; ^**d**^After TRH test; ^**e**^Three diurnal values measured at 08 h-11 h-13 h; **>> > b**: Several-fold increase compared to the mean daily prolactin.

### Discussion

The neurologic, endocrine, metabolic, neuroradiologic, and genetic investigations confirmed 4H leukodystrophy in the reported patient and ruled out possibility of any neurologic comorbidity. After excluding all non-neurologic causes of the chronic urinary incontinence [5,7] and having proven the neurogenic origin of the bladder dysfunction by the urodynamic studies [4], we concluded that the isolated micturition dysfunction had been the first and sole neurologic manifestation of the unrecognized 4H leukodystrophy for over two decades. Such a long lag between the onset of the neurogenic bladder and the subsequent neurologic symptom has not been described in Pol III-related leukodystrophy, or in any other leukodystrophies [8]. The sites responsible for the bladder dysfunction in this report seem to be the affected suprapontine brainstem tegmentum, basal ganglia, and cerebellum, along with their central afferent-efferent circuitry. Furthermore, the patient presented hypogonadotropic hypogonadism which was additionally accompanied by hypoprolactinemia and transient growth hormone deficiency. The hypoestrogenic state defined by hypogonadotropic hypogonadism was not proven to have caused urinary incontinence in this case, but such possibility should be considered in female patients with 4H leukodystrophy. The transient GH deficiency is thought to be due to lack of sex steroid priming of somatotrophs and is a novel variant of growth hormone deficiency reported in this disorder. Prolactin deficiency in 4H leukodystrophy is an intriguing observation which cannot be explained by any currently known causes of hypoprolactinemia [9]. It has been reported only in two other patients with Pol III-related leukodystrophy: originally in our first patient in 2012 [2], and recently in a Japanese patient [10]. *POLR3A* mutations have been found in these three patients. The study by Wolf et al. suggested that patients with *POLR3A* had, in general, more severe disease than patients with *POLR3B* mutations, despite the fact that disease onset was slightly earlier in the latter group [11]. This was reflected in both age at loss of supported walking and survival [11]. The role of *POLR3A* and *POLR3B* in the endocrine disturbances and the pathogenesis of these disorders still wait to be elucidated by future research [12].

## Conclusions

This is the first report providing the evidence that the myelin affection in a hypomyelinating leukodystrophy may neurologically manifest as a single symptom lasting for decades before the next neurologic decline. The fact that such a neurologic symptom can be the childhood-onset chronic urinary incontinence represents another novelty verified in the observed hypomyelinating process. The study also illustrates the complexity of the endocrine features and broadens the phenotype in Pol III-related leukodystrophy.

### Consent

Written informed consent was obtained from the patient and the parents for publication of this Case report and any accompanying images. A copy of the written consent is available for review by the Editor of this journal.

### Ethics

The study was performed in accordance with the Declaration of Helsinki and was approved by the Institutional Review Board of the Medical Faculty University of Belgrade (Ethical approval number 1832–1) and the Montreal Children's Hospital Research Ethics Board (Ethical approval number 11-105-PED).

## Abbreviations

EMG: Electromyography
FSH: Follicle-stimulating hormone
GH: Growth hormone
IGF-1: Insulin-like growth factor 1
ITT: Insulin tolerance test
LH: luteinizing hormone
LHRH: Luteinizing hormone-releasing hormone
MRI: Magnetic resonance imaging
T1W: T1-weighted
T2W: T2-weighted
TRH: Thyrotropin-releasing hormone
TSH: Thyroid-stimulating hormone
